# Alginate Hydrogel with Pluronic F-68 Enhances Cryopreservation Efficiency in Peach Germplasm

**DOI:** 10.3390/gels11120947

**Published:** 2025-11-25

**Authors:** Olena Bobrova, Milos Faltus, Viktor Husak, Jiri Zamecnik, Barbora Tunklova, Stanislav Narozhnyi, Alois Bilavcik

**Affiliations:** 1 Physiology and Cryobiology of Plants, Czech Agrifood Research Center, Drnovska 507, 16100 Prague, Czech Republic; milos.faltus@carc.cz (M.F.); jiri.zamecnik@carc.cz (J.Z.); barbora.tunklova@carc.cz (B.T.); alois.bilavcik@carc.cz (A.B.); 2 Department of Cryobiophysics, Institute for Problems of Cryobiology and Cryomedicine NAS of Ukraine, Pereyaslavska 23, 61015 Kharkiv, Ukraine; 3 Department of Biochemistry and Biotechnology, Vasyl Stefanyk Carpathian National University, 57 Shevchenka Str., 76018 Ivano-Frankivsk, Ukraine; viktor.husak@cnu.edu.ua

**Keywords:** *Prunus persica*, shoot tips, vitrification, alginate matrices, differential scanning calorimetry, plant germplasm conservation

## Abstract

The long-term conservation of *Prunus persica* (peach), a crop of significant agronomic and genetic value, remains challenging due to its recalcitrance to conventional cryopreservation methods. Low tolerance to dehydration and cryoprotectant toxicity often results in poor survival and regrowth, thereby limiting the reliability of germplasm storage. This study evaluated whether combining an alginate hydrogel matrix with Pluronic F-68 improves vitrification efficiency and post-thaw regeneration of peach shoot tips by enhancing dehydration dynamics and reducing cryo-injury. Shoot tips were immobilized in thin sodium alginate layers on aluminum foil strips, with the hydrogel providing mechanical stabilization and moderating water loss during exposure to PVS3 and subsequent liquid nitrogen immersion. To further mitigate cryoinjury, Pluronic F-68, a non-ionic surfactant with membrane-stabilizing properties, was incorporated into the system. Differential scanning calorimetry revealed that the hydrogel reached complete vitrification after 120 min in PVS3, whereas encapsulated shoot tips required 150 min for full suppression of crystallization. The optimized system achieved 71% post-cryopreservation survival and 40% regrowth, compared with 25% and 9% in non-encapsulated controls. PF-68 accelerated vitrification kinetics, lowered crystallization enthalpies, and improved post-thaw viability. These findings demonstrate that engineered hydrogel–surfactant matrices can stabilize the microenvironment during vitrification and offer a promising approach for the long-term cryopreservation of peach germplasm.

## 1. Introduction

The long-term conservation of plant genetic resources is fundamental to sustaining agricultural biodiversity, ensuring global food security, and preserving valuable traits for future breeding programs [1,2,3]. Among temperate fruit crops, *Prunus persica* (peach) occupies a position of particular importance owing to its global economic relevance, rich genetic diversity, and role as a model species within the *Prunus* genus [4,5]. Effective preservation of peach germplasm is therefore critical for both crop improvement and the maintenance of genetic heritage in global repositories.

Despite substantial progress in plant cryobiology, *P. persica* remains one of the most recalcitrant species to cryopreservation [6,7,8]. Conventional vitrification-based methods often yield low survival and regrowth rates, primarily due to the species’ pronounced sensitivity to dehydration stress, cryoprotectant toxicity, and intracellular ice formation. These physiological limitations hinder the establishment of reproducible cryobanking protocols and compromise the long-term conservation of peach genetic resources. Similar challenges have been reported for other woody perennials with complex meristem structures and limited dehydration tolerance [3,9].

Traditional vitrification solutions, such as Plant Vitrification Solution 2 (PVS2), typically rely on dimethyl sulfoxide (DMSO) as a permeating cryoprotectant [10,11]. While DMSO is effective in promoting vitrification and preventing intracellular ice, it also imposes substantial cytotoxic stress on sensitive tissues [12,13,14,15]. In *Prunus* species, DMSO exposure can be linked to oxidative damage, disruption of membrane integrity, and impaired post-thaw regeneration. Another motivation for reducing or eliminating DMSO in cryopreservation is its potential genotoxicity. Several reports in microbial, mammalian, and plant systems indicate that DMSO can induce chromosomal aberrations or DNA damage under stress-enhancing conditions [16,17]. While standard cryoprotective exposures may not reach such thresholds, the combination of dehydration, vitrification, and rewarming stresses could exacerbate these effects. Because germplasm preservation aims to maintain genetic fidelity, the adoption of DMSO-free formulations represents a prudent and low-risk alternative.

In addition to classical DMSO-based vitrification formulations such as PVS2, a range of DMSO-free strategies have been developed to minimize chemical cytotoxicity and improve tissue compatibility. These include sucrose–glycerol mixtures [10,11], gradual high-osmolarity dehydration protocols that use concentrated sugars to drive water loss, and encapsulation–dehydration methods in which water removal is regulated through alginate matrices rather than by permeating cryoprotectants [18]. Among these, Plant Vitrification Solution 3 (PVS3), composed mainly of sucrose and glycerol, represents a safer and more biocompatible alternative to DMSO-rich formulations. Sucrose promotes osmotic dehydration, stabilizes cellular membranes, and enhances the glass-forming tendency of the solution, while glycerol acts as a mild permeating agent that suppresses ice formation without causing severe chemical injury. Numerous studies have confirmed the high compatibility of PVS3 with diverse plant taxa, underscoring its suitability for delicate meristematic tissues and long-term germplasm conservation [18,19]. However, despite their improved safety, DMSO-free and encapsulation-based protocols often remain constrained by heterogeneous dehydration, limited cryoprotectant diffusion, and incomplete suppression of residual ice formation—limitations that are particularly problematic in recalcitrant woody species and complex meristem structures.

Recent advances in cryobiotechnology emphasize that beyond cryoprotectant composition, the physical microenvironment surrounding the biological specimen profoundly influences post-thaw survival. Structured polymeric matrices, particularly hydrogels and cryogels, have emerged as promising scaffolds to moderate osmotic, thermal, and mechanical stresses during cryopreservation [20,21,22,23]. Growing evidence from biomedical and environmental applications further demonstrates the capacity of polysaccharide-derived hydrogel systems to modulate mechanical, chemical, and diffusive properties in a controlled manner [24,25]. Hydrogels possess hydrated three-dimensional networks capable of regulating solute transport, buffering osmotic changes, and mechanically stabilizing embedded tissues. Cryogels, formed by cryogelation at subzero temperatures, exhibit high porosity, interconnected channels, and elasticity, properties that promote controlled dehydration and rehydration while limiting ice propagation. Their adoption in biomedical engineering, biocatalysis, and tissue engineering underscores their versatility as protective and diffusive media [26,27,28,29]. In plant cryobiology, however, the use of such materials remains underexplored.

Alginate, a naturally derived polysaccharide from brown algae, offers unique advantages for this purpose: biocompatibility, gentle gelation via ionic crosslinking, and tunable porosity [20,21,22]. Alginate hydrogels can encapsulate delicate explants, mitigating both mechanical and osmotic stress during dehydration and freezing. Their water-retaining capacity and diffusional characteristics parallel those of cryogels, positioning alginate-based systems as cryogel-inspired matrices that can bridge materials science and plant biotechnology. By providing a hydrated yet structurally stable environment, alginate matrices may buffer shoot tips against the abrupt physical and chemical changes associated with vitrification, immersion in liquid nitrogen, and subsequent thawing. To further strengthen cryoprotection, integration of functional additives such as Pluronic F-68 (PF-68) offers additional advantages [30,31,32,33]. This non-ionic triblock copolymer surfactant is widely used in mammalian and plant cell culture for its membrane-stabilizing, shear-protective, and anti-aggregation properties. Numerous reports have highlighted the beneficial effects of PF-68 as a non-ionic surfactant enhancing growth, stress tolerance, and physiological performance in plant tissue systems [34,35,36,37].

This study was designed to evaluate whether integrating a cryogel-inspired alginate hydrogel matrix with the non-ionic surfactant PF-68 can enhance the vitrification efficiency and post-thaw recovery of peach shoot tips. We hypothesized that the hydrogel framework would act as a semi-permeable, mechanically protective scaffold that modulates cryoprotectant diffusion and dehydration kinetics, while PF-68 would stabilize cell membranes, reduce cryoprotectant-induced perturbations, and promote more homogeneous water removal. To better understand the underlying thermophysical processes, differential scanning calorimetry (DSC) was employed to characterize the phase transition behavior of the hydrogel–tissue system.

The study aimed to develop and evaluate a hydrogel-assisted vitrification approach that enhances cryoprotection and regeneration efficiency of peach shoot tips while maintaining their structural and physiological integrity.

## 2. Results and Discussion

### 2.1. Thermophysical Characterization of Alginate–Sucrose Hydrogel Matrix and PVS3-Treated Shoot Tips

**Figure 1:**
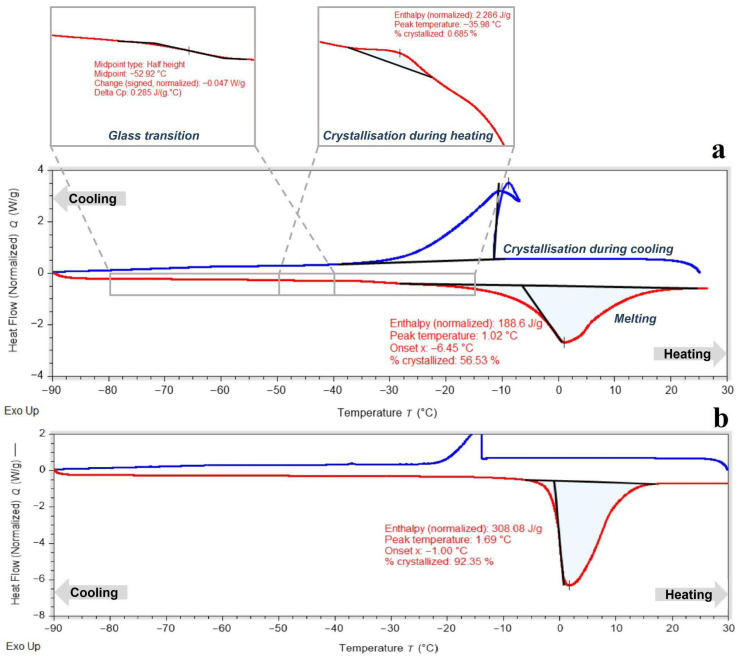
Differential scanning calorimetry (DSC) thermogram of 3% alginate hydrogel modified with 0.7 M sucrose (**a**) and 3% alginate hydrogel without modification (**b**). The DSC curve of the modified hydrogel shows the characteristic phase transitions of the alginate–sucrose hydrogel used as a coating matrix for shoot tip cryopreservation. During cooling, an exothermic peak corresponding to ice crystallization was detected with an onset at −11.5 °C and a crystallized water fraction of 54.5%. Upon reheating, a glass transition was observed near −52.9 °C, followed by a minor exothermic event at −36.0 °C (indicative of partial recrystallization) and a melting peak near 1.0 °C, with an overall crystallized water content of ~56.5%.

The use of 3% (*w*/*v*) sodium alginate is consistent with concentrations widely employed in encapsulation–dehydration and encapsulation–vitrification systems [18,19]. The alginate hydrogel used to encapsulate peach shoot tips was formulated with 0.7 M sucrose, matching the final step of the sucrose preculture treatment. This ensured osmotic compatibility between the encapsulating matrix and the preconditioned plant tissue. DSC analysis of the 3% alginate hydrogel containing sucrose revealed a mixed amorphous–crystalline structure (Figure 1a). A pronounced exothermic crystallization peak was observed near −11 °C, corresponding to approximately 55% crystallized water. A glass transition was detected around −53 °C, indicating the formation of an amorphous phase upon cooling; however, this glassy phase was unstable, and upon heating above the glass transition temperature, crystallization of the supercooled water fraction occurred. The considerable amount of crystallized water and unstable amorphous structure highlight the need for further hydrogel modification with cryoprotective solutes to suppress crystallization and promote a fully vitrified hydrogel capable of retaining a high fraction of unfrozen water at low temperatures. For comparison, the DSC thermogram of pure 3% alginate (without sucrose) is shown in Figure 1b. Unmodified alginate displayed a melting peak with a very high crystallized water fraction, confirming that it lacks intrinsic vitrification capacity. The marked lowering of the crystallization temperature and the appearance of a partial amorphous phase after sucrose addition (Figure 1a) highlight sucrose’s essential role in improving hydrogel vitrification behavior and justify its inclusion in the encapsulation formulation.

The classical droplet vitrification method involves gradual dehydration and cryoprotectant penetration using a loading solution (LS), followed by exposure to a vitrification solution [19,38]. Typically, DMSO-based solutions are used because of their rapid permeability [39]; however, in this study, we employed PVS3 (a DMSO-free mixture of glycerol and sucrose) to examine its vitrification capacity. DSC was used to determine the minimum exposure time required for sufficient tissue dehydration and stable amorphous matrix formation during both cooling and warming. This approach also allowed us to monitor dehydration dynamics in encapsulated and non-encapsulated shoot tips.

**Figure 2:**
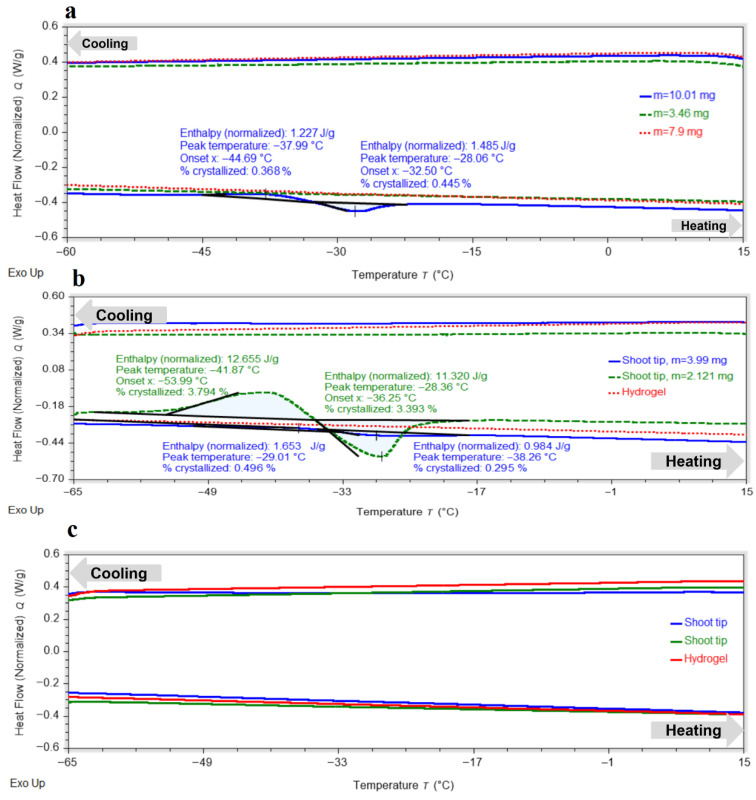
Differential scanning calorimetry (DSC) thermograms of peach shoot tips and alginate hydrogel matrix after incubation in PVS3. (**a**) Shoot tips exposed to PVS3 without hydrogel for 80 min exhibited complete vitrification during cooling, with no exothermic peaks detected. Only minor transitions were observed in a few samples upon heating (crystallization near −38 °C; melting −28 °C), and the proportion of crystallized water remained below 0.5%. (**b**) After 120 min in PVS3, the hydrogel matrix exhibited no exothermic or endothermic transitions, indicating full vitrification. Encapsulated shoot tips displayed only small heating peaks, corresponding to limited crystallization of supercooled water followed by melting (<4% crystallized water). (**c**) After 150 min in PVS3, all residual thermal effects disappeared, confirming complete vitrification of both the alginate hydrogel and the enclosed shoot tips.

Shoot tips exposed to PVS3 without hydrogel for 80 min exhibited complete vitrification during cooling, with no exothermic peaks detected (Figure 2a). In most samples, no exothermic or endothermic transitions were observed upon heating, indicating the absence of ice crystallization or melting. Only minor transitions were detected in a few samples, with crystallization onset around −44.7 °C and melting near −28.1 °C. The proportion of crystallized water remained consistently below 0.5%, demonstrating that most of the water in the tissues had been converted into an amorphous glassy state during cooling, with only a small metastable fraction capable of recrystallizing upon warming at low warming rate.

For the hydrogel matrix, at least 120 min in PVS3 were required to achieve full vitrification (Figure 2b), and 150 min were necessary to ensure a completely stable amorphous state in the encapsulated peach shoot tips (Figure 2c). These exposure durations were selected based on preliminary DSC screening tests in which shorter PVS3 treatments produced detectable crystallization peaks. After 120 min, the hydrogel exhibited no exo- or endothermic transitions, while the shoot tips showed only small peaks upon heating, corresponding to limited crystallization of supercooled water followed by melting (<4% crystallized water). After 150 min, all residual thermal effects disappeared, confirming the formation of a fully vitrified state in both hydrogel and tissues. The shorter vitrification time of the hydrogel relative to the embedded shoot tips reflects differences in mass-transfer dynamics. While the alginate matrix equilibrates rapidly with PVS3, cryoprotectant penetration into the multilayered meristematic tissue occurs more slowly, requiring prolonged exposure to achieve complete vitrification. The hydrogel does not impede PVS3 transport; rather, it moderates diffusion and reduces osmotic shock by smoothing the concentration gradient. PF-68 may further enhance penetration by improving surface wettability and decreasing interfacial tension, although this effect was not quantified in the present study.

**Figure 3:**
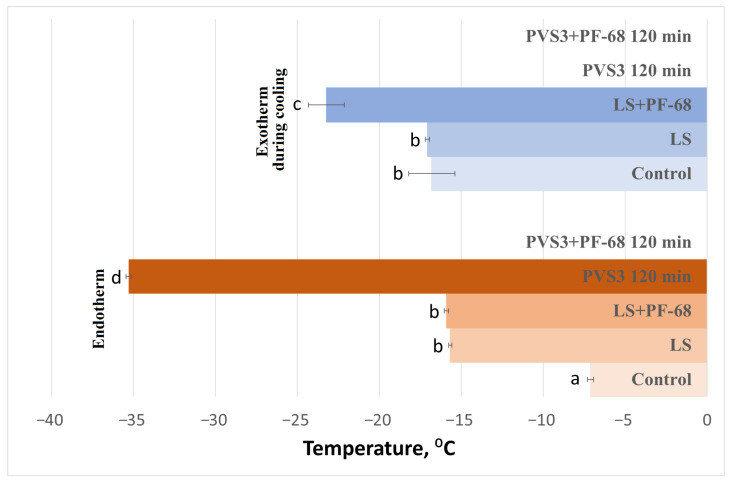
Crystallization and melting onset temperatures in alginate–sucrose hydrogels during cryoprotectant treatment. Onset temperatures (°C) of crystallization during cooling and melting during heating were determined by DSC in 3% alginate hydrogels containing 0.7 M sucrose after incubation in the loading solution (LS) and Plant Vitrification Solution 3 (PVS3) for 120 min, with or without Pluronic F-68 (PF-68). Each bar represents mean ± SE (*n* = 9); different letters indicate significant differences (*p* < 0.05). The progressive decrease in crystallization and melting temperatures reflects enhanced dehydration and vitrification of the hydrogel, with faster suppression of ice formation in the presence of PF-68.

**Figure 4:**
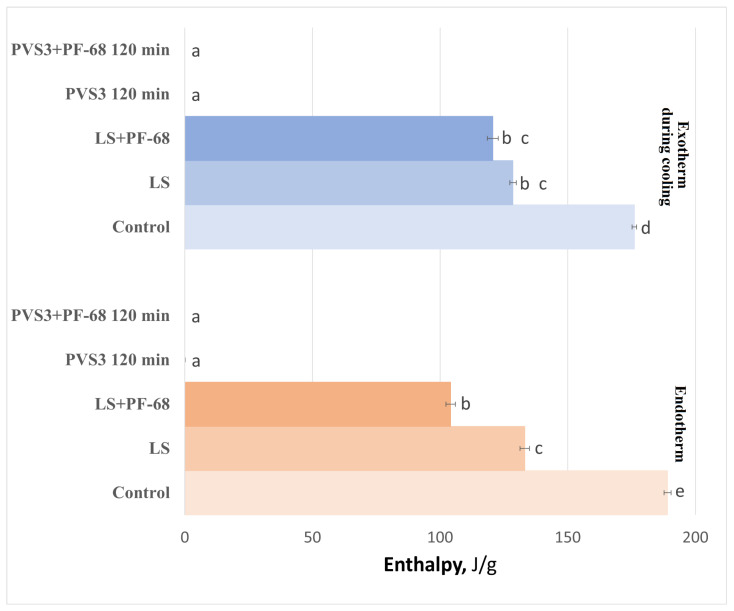
Crystallization and melting enthalpies in alginate–sucrose hydrogels during cryoprotectant treatment. Normalized enthalpies (J·g^−1^) corresponding to crystallization and melting transitions were determined by DSC for hydrogels incubated in LS and PVS3 (120 min), with or without Pluronic F-68 (PF-68). Values are expressed as mean ± SE (*n* = 9); different letters indicate significant differences (*p* < 0.05). Both crystallization and melting enthalpies declined markedly after exposure to cryoprotectants, indicating progressive vitrification of the hydrogel matrix. Hydrogels containing PF-68 exhibited significantly lower enthalpy values, confirming accelerated water immobilization and reduced ice formation.

The influence of the surfactant PF-68 on water-state dynamics in the hydrogel was also examined during exposure to LS and PVS3, reflecting the real conditions of cryopreservation experiments. Progressive changes in the hydrogel’s thermal behavior were observed during sequential exposure to LS and PVS3. Crystallization and melting onset temperatures decreased with increasing incubation time (Figure 3), accompanied by a marked reduction in enthalpy values (Figure 4), indicating enhanced dehydration and shift from a partially crystalline to a predominantly amorphous state. After 120 min in PVS3, the hydrogel was almost fully vitrified, showing no crystallization during cooling and only a minimal melting peak during heating (<0.1% crystallized water). The addition of PF-68 accelerated this process: hydrogels containing the surfactant exhibited significantly lower crystallization temperatures after only 20 min in LS and significantly reduced melting enthalpies compared with hydrogels without PF-68 (*p* < 0.05). This acceleration is likely to result from PF-68 promoting more effective solute diffusion and disrupting hydrogen-bonded water clusters within the alginate network.

These results clearly demonstrate that incubation with cryoprotectants and surfactant modification act synergistically to enhance the vitrification capacity of alginate-based hydrogels. PF-68-enriched matrices facilitated faster suppression of ice formation and more complete water immobilization—both essential for preventing ice-induced injury in encapsulated tissues.

**Figure 5:**
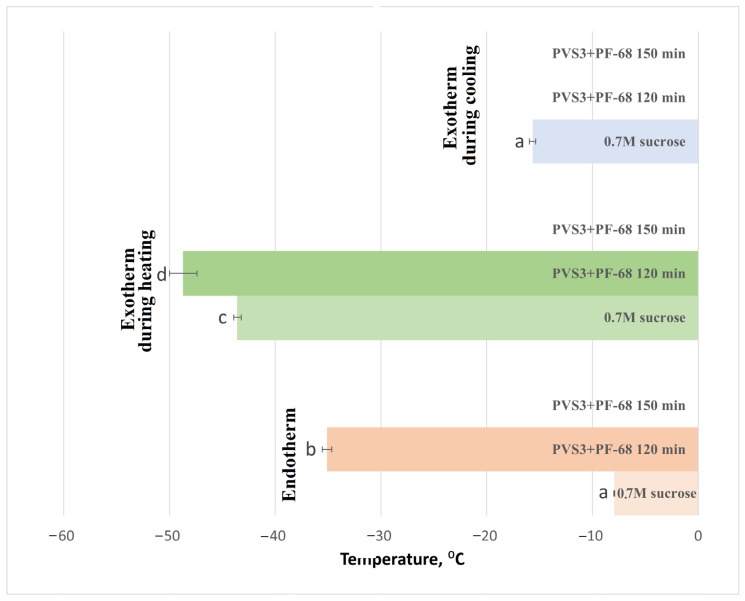
Crystallization and melting onset temperatures in peach (*Prunus persica*) shoot tips after exposure to PVS3 + Pluronic F-68 (PF-68). Onset temperatures (°C) of crystallization during cooling and heating, and melting during reheating, were determined by DSC following 120 and 150 min of incubation in PVS3 + PF-68, compared with 0.7 M sucrose (preculture control). Data represents mean ± SE (*n* = 9); different letters indicate significant differences (*p* < 0.05). The disappearance of crystallization and melting peaks with prolonged incubation reflects complete vitrification of shoot tip tissues within the hydrogel matrix.

**Figure 6:**
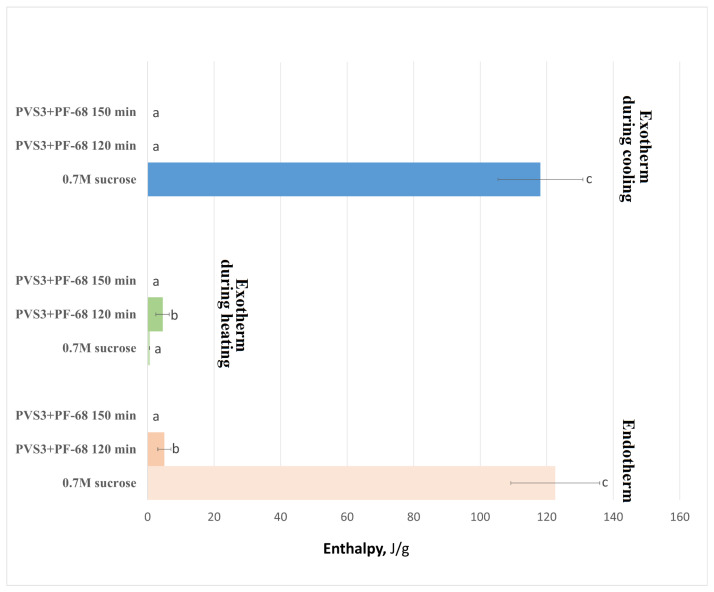
Crystallization and melting enthalpies in peach shoot tips after exposure to PVS3 + Pluronic F-68 (PF-68). Normalized enthalpies (J·g^−1^) corresponding to crystallization during cooling and heating, and melting, were determined by DSC in shoot tips treated for 120 and 150 min in PVS3 + PF-68, compared with those in 0.7 M sucrose (control). Values represent mean ± SE (*n* = 9); different letters indicate significant differences (*p* < 0.05). The marked reduction in crystallization and melting enthalpies in PVS3 + PF-68 samples demonstrate progressive dehydration and water immobilization, resulting in a stable amorphous state at 150 min.

DSC analysis of hydrogel-encapsulated shoot tips saturated with PVS3 + PF-68 revealed a clear progression toward complete vitrification with increasing incubation time, as indicated by decreasing crystallization and melting temperatures (Figure 5) and enthalpies (Figure 6), followed by the disappearance of these thermal transitions altogether. This trend reflects increased dehydration efficiency and vitrification over time. It is noteworthy that a minor exothermic effect during heating was observed only in control hydrogels (Figure 1), reflecting the instability of their amorphous state. After LS incubation, this effect was no longer detectable, and therefore only two thermal events (cooling exotherm and melting endotherm) are shown in Figure 3 and Figure 4. In shoot tips (Figure 5 and Figure 6), a small metastable glassy fraction remained after sucrose preculture and 120 min PVS3 exposure, producing minor exothermic crystallization peaks during warming.

The combination of alginate and sucrose produced a semi-vitrified hydrogel capable of retaining large amounts of unfrozen water. This property is advantageous for cryopreservation because it limits ice propagation, moderates dehydration kinetics, and maintains a flexible, hydrated microenvironment around shoot tips. Such behavior resembles that of cryogels, where interconnected porous networks and controlled water confinement prevent mechanical stress. Consequently, the alginate–sucrose matrix functions as a cryogel-inspired protective medium, stabilizing vitrification and enhancing tissue integrity during freezing and thawing.

Together, these findings demonstrate that the combined use of sucrose-modified alginate, PVS3, and PF-68 creates an effective cryogel-like microenvironment that maintains shoot tips in a metastable amorphous state. This vitrification stability minimizes ice nucleation during both cooling and rewarming, providing a thermophysically robust platform for the long-term cryopreservation of peach germplasm.

### 2.2. Post-Cryopreservation Survival and Regrowth

**Figure 7:**
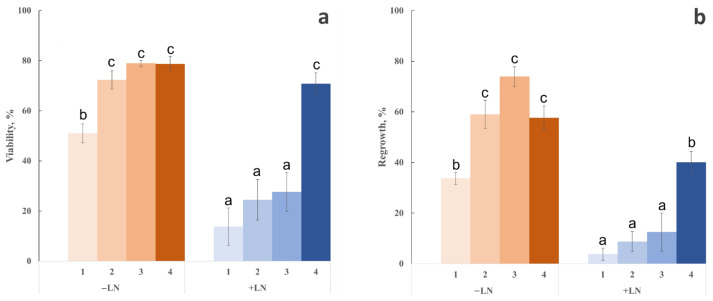
Post-thaw survival and regrowth of *Prunus persica* shoot tips after vitrification using different cryoprotection strategies. (**a**) Viability (%) of shoot tips two weeks after treatment. (**b**) Regrowth (%) was assessed four weeks after treatment. Groups: (1) classical droplet vitrification (PVS3, 80 min); (2) droplet vitrification with Pluronic F-68 (80 min); (3) hydrogel matrix + PVS3 (120 min); (4) hydrogel matrix + PVS3 (150 min). −LN: samples subjected only to cryoprotectant treatment without freezing; +LN: samples vitrified and stored in liquid nitrogen. Values represent mean ± SE (*n* ≥ 30); different letters indicate significant differences (*p* < 0.05).

To evaluate the biological efficiency of the developed cryoprotection strategy, we compared the modified hydrogel-based vitrification approach with the classical droplet vitrification method. In addition to the conventional PVS3 treatment, we assessed the individual effect of the non-ionic surfactant PF-68 and the influence of the alginate hydrogel matrix. Both the effect of the cryoprotectant exposure and dehydration step (−LN, non-frozen) and the impact of vitrification and rewarming (+LN, liquid nitrogen exposure) were analyzed. Viability of shoot tips was scored after two weeks of recovery (Figure 7a), and regrowth (elongation and leaf development) was recorded after four weeks (Figure 7b).

Exposure of peach shoot tips to PVS3 for 80 min (Group 1, classical droplet vitrification without PF-68) resulted in a pronounced reduction in viability and regrowth compared with non-treated controls (≈100% regrowth under standard in vitro conditions). Viability decreased to approximately 51% and regrowth to 34%, indicating substantial osmotic and chemical stress associated with PVS3 exposure. In Group 2, where PF-68 was included in both LS and PVS3, viability increased significantly to 72%, and regrowth reached 59% (*p* < 0.05). After cryopreservation (+LN), survival and regrowth decreased in both groups, reflecting the additional stress associated with freezing and rewarming. However, PF-68 nearly doubled both parameters compared with Group 1. These results demonstrate that PF-68 effectively mitigates cryoprotectant toxicity and mechanical stress, likely through membrane stabilization and reduction in osmotic injury during vitrification and thawing.

Shoot tips encapsulated in the modified hydrogel matrix (Groups 3 and 4) exhibited high viability even before freezing (−LN). After 120 or 150 min of incubation in PVS3, their survival and regrowth were comparable to or higher than those in Group 2 (classical vitrification with PF-68). Post-thaw survival and regrowth varied depending on the duration of PVS3 exposure. After 120 min of incubation, viability significantly decreased following LN exposure compared with unfrozen controls, with regrowth reaching only about 13%. Extending the incubation to 150 min markedly improved cryotolerance, yielding 71% viability (Figure 7a) and approximately 40% regrowth (Figure 7b). These values were statistically like unfrozen controls for classical droplet vitrification (Group 1), indicating that optimized hydrogel-mediated vitrification effectively protects against cryoinjury.

**Figure 8:**
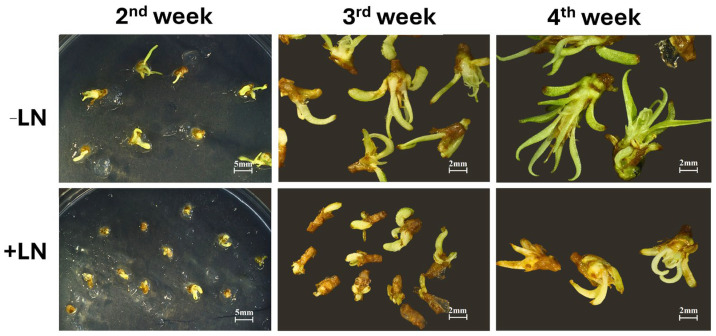
Regrowth of hydrogel-encapsulated peach shoot tips following vitrification and cryopreservation. Representative images show regrowth progression after 2, 3, and 4 weeks of culture following the cryoplate-vitrification procedure with hydrogel encapsulation. Upper panels: non-frozen (−LN) shoot tips; lower panels: cryopreserved (+LN) shoot tips. Gradual restoration of shoot elongation and leaf development was observed in cryopreserved samples after 150 min PVS3 exposure, indicating successful recovery and viability.

Regeneration dynamics were also affected by cryopreservation status. While unfrozen shoot tips showed rapid bud elongation and leaf development, cryopreserved samples required more time to resume active growth, and regenerated plantlets remained visibly smaller after four weeks (Figure 8). This delayed post-thaw regrowth likely reflects residual stress associated with vitrification and rewarming, even in optimally protected tissues.

Importantly, these biological outcomes are fully consistent with the thermophysical data presented in Section 2.1. DSC analysis demonstrated that 150 min of exposure to PVS3 in combination with PF-68 resulted in complete vitrification of both the hydrogel matrix and the encapsulated shoot tips, with no detectable crystallization or melting transitions. The absence of residual ice corresponds directly to the observed high post-thaw survival and regrowth rates. Conversely, shorter equilibration periods (120 min) were associated with partial or metastable vitrification, which was reflected in reduced viability and slower recovery. These results highlight the strong link between vitrification dynamics and biological performance, emphasizing the critical importance of achieving a stable amorphous state to maximize cryotolerance.

Overall, our findings demonstrate that combining PF-68 with an alginate hydrogel matrix significantly enhances dehydration kinetics, suppresses ice formation, and improves post-thaw regeneration of peach shoot tips. The integration of thermophysical insights with biological outcomes provides a robust foundation for the development of reproducible, high-efficiency cryopreservation protocols for recalcitrant woody species.

### 2.3. Mechanistic Model of Hydrogel-Mediated Cryoprotection

The integration of thermophysical analyses and post-thaw recovery data allows us to propose a mechanistic framework in which the alginate–sucrose–Pluronic hydrogel functions as a cryogel-inspired vitrification scaffold, creating a multi-layered protective microenvironment around peach shoot tips during ultra-low temperature exposure. This system may enhance vitrification efficiency and mitigate cryo-injury by concurrently moderating osmotic, thermal, and mechanical stress factors—which are typically difficult to control in classical droplet vitrification systems. Similar conceptual frameworks have been proposed for mammalian and algal cells, where structured polymeric matrices can stabilize the microenvironment during freezing [20,21,22,23,40].

1. Controlled Cryoprotectant Diffusion and Dehydration Dynamics

The porous, hydrated architecture of the alginate matrix likely acts as a semipermeable interface, enabling gradual penetration of cryoprotectant components and controlled tissue dehydration. This moderate diffusion is particularly relevant for highly sensitive meristematic peach tissues, which display poor tolerance to abrupt osmotic changes. Our DSC analyses revealed that progressive equilibration in the matrix led to a gradual reduction in crystallization enthalpy, approaching zero after 150 min of exposure to PVS3, suggesting complete vitrification. Such controlled dehydration is consistent with previous observations that slower cryoprotectant penetration reduces osmotic shock and volumetric strain in cells [41]. This may allow cellular membranes, organelles, and the cytoskeleton to adapt to hyperosmotic environments more gradually, reducing the risk of membrane rupture or cytorrhysis.

2. Suppression of Ice Nucleation and Water Mobility

A critical determinant of cryopreservation success is the degree to which freezable water is immobilized before cooling. Within the alginate–sucrose hydrogel, nanoscale confinement and hydrogen bonding networks appear to restrict water mobility, thereby stabilizing residual water in an amorphous state. This observation is in line with earlier studies showing that hydrogels can shift water into a bound or vitrified state, thereby reducing ice crystal formation [42,43]. Sucrose plays a dual role in this process: it elevates the glass transition temperature and forms hydrogen-bonded protective shells around macromolecules, further stabilizing the amorphous phase. Consistent with these mechanisms, DSC thermograms of hydrogel-treated samples demonstrated complete vitrification with negligible crystallization during cooling and rewarming. This likely contributes to the suppression of both heterogeneous and homogeneous ice nucleation, minimizing intracellular mechanical damage and recrystallization-associated necrosis, a well-documented cause of post-thaw failure in plant vitrification protocols [11,44].

3. Membrane Stabilization through Surfactant-Mediated Protection

Surfactant PF-68 introduces another layer of protection, likely by lowering interfacial tension and stabilizing cellular membranes during critical dehydration and cooling steps. Non-ionic surfactants of this class are known to reduce membrane fusion, stabilize lipid bilayers, and mitigate shear or osmotic stress [30,31,32,33]. In our system, PF-68 appeared to accelerate equilibration, as evidenced by the more rapid reduction in crystallization onset temperatures and enthalpies. This effect may be explained by improved wettability and enhanced cryoprotectant diffusion across tissue interfaces. In addition, PF-68 may interact with phospholipid membranes, buffering them against phase transitions and dehydration-induced perturbations, which are major contributors to cryo-injury in plant meristems. Although PF-68 is widely reported to reduce interfacial tension, stabilize membranes, and improve cryoprotectant equilibration, these effects were not directly measured in the present system. Future studies incorporating surface tension assays, membrane integrity markers, and solute diffusion measurements will be essential to experimentally validate the proposed surfactant-mediated mechanisms.

4. Integrated Cryoprotection and System Stabilization

These three mechanisms, involving controlled cryoprotectant diffusion, suppression of water mobility and ice formation, and membrane stabilization, are likely to act synergistically to create a stable vitrification microenvironment both inside and around the tissue. Such a microenvironment may substantially reduce osmotic shock, mechanical injury, and intracellular ice damage. This integrative protection is reflected in the markedly improved post-thaw viability and regrowth observed after 150 min of PVS3 exposure in the presence of PF-68. The overall behavior of the hydrogel matrix closely resembles engineered cryogels used in biomedical cryobiology, where macroporous polymer networks improve freezing tolerance by regulating mass transfer and thermal behavior [45,46].

Although the three synergistic mechanisms proposed—controlled cryoprotectant diffusion, suppressed ice nucleation, and membrane stabilization—are strongly supported by DSC thermophysical behavior and post-thaw biological outcomes, the present study did not include complementary biochemical or ultrastructural assays. Future research should incorporate indicators of membrane integrity, antioxidant responses, and ultrastructural examination of organelles by TEM to directly visualize hydrogel-mediated protection. These analyses would provide valuable molecular and cellular validation of the mechanistic model presented here.

In this study, only PF-68 was evaluated; however, other non-ionic surfactants such as Tween-20, Triton X-100, and related poloxamers may also offer beneficial effects by stabilizing membranes, reducing osmotic shock, and improving cryoprotectant equilibration in cryosensitive tissues. Nevertheless, PF-68 has a particularly well-documented record of safe and effective use in both plant and mammalian cell systems, where it reduces shear stress, moderates membrane injury, and enhances post-thaw viability [30,31].

Recent studies have explored a variety of polysaccharide-based hydrogels—including chitosan, pectin, gellan gum, and cellulose derivatives—but alginate remains among the most widely used encapsulation materials for the cryopreservation of plant germplasm, owing to its biocompatibility, mild gelation conditions, and tunable diffusional properties [18,19]. From a practical perspective, both alginate and PF-68 are relatively inexpensive and readily accessible compared with specialized commercial cryopreservation kits. Although the protocol introduces an encapsulation step, it requires no specialized equipment beyond standard tissue culture materials, making the method feasible for routine use in germplasm repositories. These considerations underscore the potential of the proposed platform not only for improving cryopreservation outcomes but also for enabling cost-effective implementation in gene bank operations.

Together, the alginate–PF-68 system therefore offers a biocompatible, modular, and technically accessible platform that integrates polymer-based microenvironment engineering with surfactant-mediated membrane stabilization. These combined properties make it highly suitable for integration into cryopreservation workflows for recalcitrant woody species and genetically valuable germplasm.

## 3. Conclusions

This study demonstrates that cryogel-inspired alginate matrices co-formulated with Pluronic F-68 markedly improve vitrification efficiency and post-thaw recovery of *Prunus persica* shoot tips. By moderating cryoprotectant diffusion, stabilizing water in an amorphous state, and enhancing membrane robustness, the alginate–PF-68 system provides a physically and physiologically protective microenvironment that significantly reduces cryo-injury.

Conceptually, our findings support the broader idea that engineering the physical microenvironment of plant tissues—rather than modifying cryoprotectant chemistry alone—can substantially enhance cryopreservation outcomes, especially for recalcitrant meristems. Nevertheless, certain limitations must be acknowledged. The study did not encompass all genotype × treatment combinations, and only early post-thaw regrowth was evaluated. Future work should expand genotype testing, optimize matrix formulations, and assess long-term plant stability.

Despite these limitations, the hydrogel-assisted vitrification approach represents a robust, reproducible, and biocompatible method for preserving sensitive germplasm and may be adapted to other woody or recalcitrant species.

## 4. Materials and Methods

### 4.1. Plant Material

In vitro–grown plants of six *Prunus persica* L. genotypes—‘Flame Prince’, ‘Vate 1543’, ‘Guglielmina’, ‘Nectared 4’, ‘Rich May’, and ‘Delice’—from the in vitro collection of the Czech Agrifood Research Center were used as the source of explants. Shoot cultures, 3–4 weeks old, were maintained on standard Quoirin and Lepoivre (QL) basal medium [47] containing 3% (*w*/*v*) sucrose and agar 7 g·L^−1^, with the pH adjusted to 6.2. The medium was supplemented with 0.4 mg·L^−1^ 6-benzylaminopurine (BAP) and 0.01 mg·L^−1^ 1-naphthaleneacetic acid (NAA). Cultures were grown under controlled conditions in a growth chamber at 25 ± 2 °C with a 16 h photoperiod (photosynthetic photon flux density of approximately 40–50 µmol m^−2^·s^−1^).

Apical shoot tips, approximately 2–3 mm in length, were aseptically excised from actively growing in vitro plantlets under a stereomicroscope. Only healthy, uniformly developed shoots with visible meristematic domes were selected to ensure consistency in explant quality across all treatments.

### 4.2. Cryoprotection

**Figure 9:**
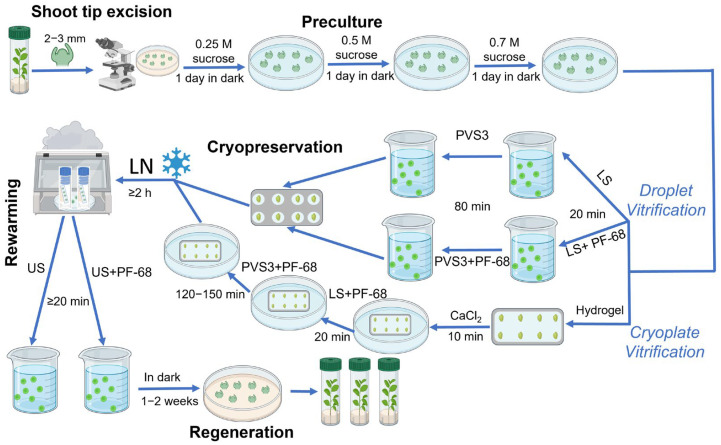
Experimental workflow for cryopreservation of peach (*Prunus persica*) shoot tips using droplet- and cryoplate-vitrification protocols. The process includes shoot tip excision, sucrose preculture, cryoprotection with loading solution (LS) and Plant Vitrification Solution 3 (PVS3), each applied with or without the surfactant Pluronic F-68 (PF-68), vitrification in liquid nitrogen (LN), rewarming in unloading solution (US), and post-thaw regeneration on QL medium.

Excised shoot tips were precultured for three consecutive days in liquid QL medium (standard QL medium without the agar) supplemented with sucrose at gradually increasing concentrations of 0.25 M, 0.5 M, and 0.7 M at 25 °C in the dark (Figure 9). The preculture medium was renewed every 24 h to prevent sugar depletion and to promote progressive osmotic adjustment before exposure to cryoprotectants.

Following preculture, shoot tips were subjected to a cryoprotection protocol consisting of exposure to LS—followed by PVS3.

Loading solution (LS): Explants were treated with a solution containing 0.4 M sucrose and 2 M glycerol prepared in H_2_O for 20 min at room temperature.

Plant Vitrification Solution (PVS3): Subsequently, shoot tips were transferred to PVS3, composed of 40% (*w*/*v*) glycerol and 40% (*w*/*v*) sucrose in H_2_O. Exposure time to PVS3 was 80 min for the droplet-vitrification procedure and 120–150 min for the modified cryoplate-vitrification method, both conducted at room temperature.

Both LS and PVS3 treatments were applied either in the absence (control) or presence of the non-ionic surfactant PF-68 (Sigma-Aldrich, Saint Louis, MO, USA) at concentrations of 0.05% (*w*/*v*) or 0.1% (*w*/*v*), respectively. The inclusion of PF-68 was evaluated for its membrane-stabilizing and cryoprotective properties. This concentration range was selected based on values commonly used in cell culture systems, where PF-68 is recognized as biocompatible and non-toxic to living cells [30,48].

### 4.3. Alginate Encapsulation

In a parallel set of treatments, shoot tips were encapsulated in alginate hydrogel matrices before cryoprotection. Explants were immersed in a sterile solution of 3% (*w*/*v*) sodium alginate supplemented with 0.7 M sucrose and carefully positioned onto aluminum foil strips. Controlled gelation was achieved by contacting the alginate layer with 50 mM CaCl_2_ solution supplemented with 0.7 M sucrose (to prevent sucrose loss from the alginate matrix) for 10 min at room temperature (22–24 °C). This process produced a thin, uniform hydrogel coating surrounding each explant. The encapsulated shoot tips were subsequently subjected to the LS and PVS3 treatments described above (Figure 9).

### 4.4. Cooling and Thawing

Prepared shoot tips, both alginate-coated and non-coated, were aligned on sterile aluminum foil strips and rapidly plunged into liquid nitrogen (−196 °C) for vitrification. Samples were stored for at least 2 h before rewarming. Thawing was carried out by direct immersion of the foil strips into an unloading solution (US) consisting of 0.7 M sucrose in H_2_O, with or without 0.01% (*w*/*v*) PF-68, for a minimum of 20 min at room temperature (Figure 9). A lower PF-68 concentration (0.01% *w*/*v*) was used in the unloading solution to promote membrane rehydration while minimizing prolonged surfactant exposure, which could interfere with early post-thaw recovery. In contrast, higher concentrations (0.05–0.1% *w*/*v*) in LS and PVS3 were required to stabilize membranes during the most osmotically and thermally stressful stages of cryoprotection. This concentration gradient reflects standard practice in plant tissue culture, where surfactant levels are reduced during recovery phases to minimize potential interference with metabolic reactivation.

### 4.5. Post-Thaw Recovery

Following thawing, shoot tips were transferred onto a semisolid *Prunus* Recovery Medium consisting of standard QL medium without NH_4_^+^ to support regrowth [49]. After two weeks of initial culture, explants were transferred to standard medium and incubated under the same conditions as the stock cultures.

Survival was evaluated after two weeks, based on the presence of green, turgid tissues. Regrowth, defined as shoot elongation and leaf development, was recorded over four weeks. Regrowth percentage was calculated relative to the total number of thawed shoot tips. Each treatment included at least three independent biological replicates, with a minimum of 30 explants per treatment.

### 4.6. Differential Scanning Calorimetry (DSC)

Low-temperature phase transitions in alginate hydrogels and peach shoot tips were analyzed using a Discovery X3 differential scanning calorimeter (TA Instruments, New Castle, DE, USA). Samples were hermetically sealed in standard aluminum pans and subjected to controlled cooling and heating cycles over a temperature range from −90 °C to +25 °C. A constant cooling/heating rate of 10 °C·min^−1^ was used for all experiments to ensure comparability among samples. Each analysis was performed in triplicate to ensure reproducibility. Thermograms were processed using TRIOS software V5.6 (TA Instruments) to determine the onset and completion temperatures of crystallization and melting events, as well as to estimate the proportion of crystallized water in each sample.

### 4.7. Statistical Analysis

All analyses were performed in R (version 4.3.1). Because proportional outcomes showed deviations from normality and/or heteroscedasticity, differences among groups were evaluated using the Kruskal–Wallis rank-sum test (non-parametric ANOVA). When the omnibus test was significant (α = 0.05), pairwise post hoc comparisons were conducted with Dunn’s test; adjusted *p*-values were reported, and results were considered statistically significant at *p* < 0.05. Data in figures are presented as mean ± SE with sample sizes indicated (e.g., *n* = 9), and distinct lowercase letters above symbols/bars denote groups that differ significantly within each endpoint. All inferential tests were two-tailed with a significance threshold of *p* < 0.05, thereby evaluating the possibility of both positive and negative deviations from the null hypothesis.

### 4.8. Experimental Design Note

The six genotypes were included to provide biological replication across diverse genetic backgrounds rather than to compare cultivar-specific responses. The study was not powered to detect genotype effects, and the genotype-by-treatment layout was unbalanced. The primary objective was to assess the effects of hydrogel encapsulation, PF-68, vitrification exposure time, and liquid nitrogen treatment on shoot tip survival and regrowth. Because not all treatment combinations were tested for every genotype, genotype was modeled as a random blocking factor rather than as a fixed effect of interest.

## Data Availability

Data is contained within the article.

## Abbreviations

The following abbreviations are used in this manuscript:

DSC	Differential Scanning Calorimetry
PVS	Plant Vitrification Solution
LN	Liquid nitrogen
PF-68	Pluronic F-68
LS	Loading Solution
US	Unloading Solution
DMSO	Dimethyl sulfoxide
SE	Standard errors
